# Event Networks and the Identification of Crime Pattern Motifs

**DOI:** 10.1371/journal.pone.0143638

**Published:** 2015-11-25

**Authors:** Toby Davies, Elio Marchione

**Affiliations:** 1 Department of Civil, Environmental and Geomatic Engineering, University College London, London, United Kingdom; 2 Department of Security and Crime Science, University College London, London, United Kingdom; 3 Centre for Advanced Spatial Analysis, University College London, London, United Kingdom; Tianjin University, CHINA

## Abstract

In this paper we demonstrate the use of network analysis to characterise patterns of clustering in spatio-temporal events. Such clustering is of both theoretical and practical importance in the study of crime, and forms the basis for a number of preventative strategies. However, existing analytical methods show only that clustering is present in data, while offering little insight into the nature of the patterns present. Here, we show how the classification of pairs of events as close in space and time can be used to define a network, thereby generalising previous approaches. The application of graph-theoretic techniques to these networks can then offer significantly deeper insight into the structure of the data than previously possible. In particular, we focus on the identification of network motifs, which have clear interpretation in terms of spatio-temporal behaviour. Statistical analysis is complicated by the nature of the underlying data, and we provide a method by which appropriate randomised graphs can be generated. Two datasets are used as case studies: maritime piracy at the global scale, and residential burglary in an urban area. In both cases, the same significant 3-vertex motif is found; this result suggests that incidents tend to occur not just in pairs, but in fact in larger groups within a restricted spatio-temporal domain. In the 4-vertex case, different motifs are found to be significant in each case, suggesting that this technique is capable of discriminating between clustering patterns at a finer granularity than previously possible.

## Introduction

Many fields of study involve the analysis of sets of events which occur at distinct locations in space and time. These arise frequently in epidemiology [1–3], in which events typically represent cases of disease, while recent research has also begun to examine the occurrence of criminal incidents in a similar manner [4–6]. In both cases, the identification of patterns can offer insight into the underlying generative processes, while also suggesting potential preventative strategies.

Of particular interest in these contexts is the phenomenon of space-time clustering, whereby events tend to occur close to each other in both space and time. This corresponds to interaction between the spatial and temporal distributions of events: in particular, the tendency of events which are near in time to also be near in space (and *vice versa*). It should be noted that this notion of clustering is distinct from its meaning in relation to the partitioning of data into groups: the issue of interest here is simply the relationship between spatial and temporal proximity.

For real-world phenomena, such a relationship is of particular significance because it implies that the spatial distribution of events at any given time is dependent upon their previous locations: events tend to happen in the near spatial vicinity of recent events (*i.e.* those that are near temporally). This suggests a causal relationship between events, and therefore that risk is communicable, in some sense, across space.

Empirical research has demonstrated that clustering of this form can be observed for a range of crimes [5–7]. On one hand, this has provided insight into criminal targeting behaviour, and has motivated a number of theoretical developments [8–10]. However, it also has significant practical implications. The presence of clustering implies that crime is, to some extent, predictable: the risk of victimisation is temporarily increased in the vicinity of a recent incident. This is one of the principles which forms the basis for the emerging field of ‘predictive policing’ [11, 12], in which police resources are directed towards areas where crime is anticipated to occur in the near future.

In terms of urban crime prevention, therefore, it is apparent that understanding patterns of space-time clustering is a key issue. Furthermore, recent research has shown that clustering is also present in data for several non-urban criminal phenomena, such as maritime piracy [13, 14] and insurgent activity [15, 16]. It has therefore been speculated that event prediction may also be feasible in these contexts; however, whether the patterns are sufficiently similar is not currently known.

The measurement of space-time clustering is distinct from its analysis in space or time only [17], since the primary concern is the interaction between the two dimensions. Existing approaches are based on comparison between the observed separation of events and that which would be expected if their spatial and temporal distributions were independent. Common methods involve the pair-wise comparison of events, where pairs are classified as being ‘close pairs’ if they lie within some specified thresholds in both space and time [18, 19], or if they are nearest neighbours in both space and time [20]. The number of close pairs is then compared against that which would be expected if locations and timings were independent.

**Fig 1:**
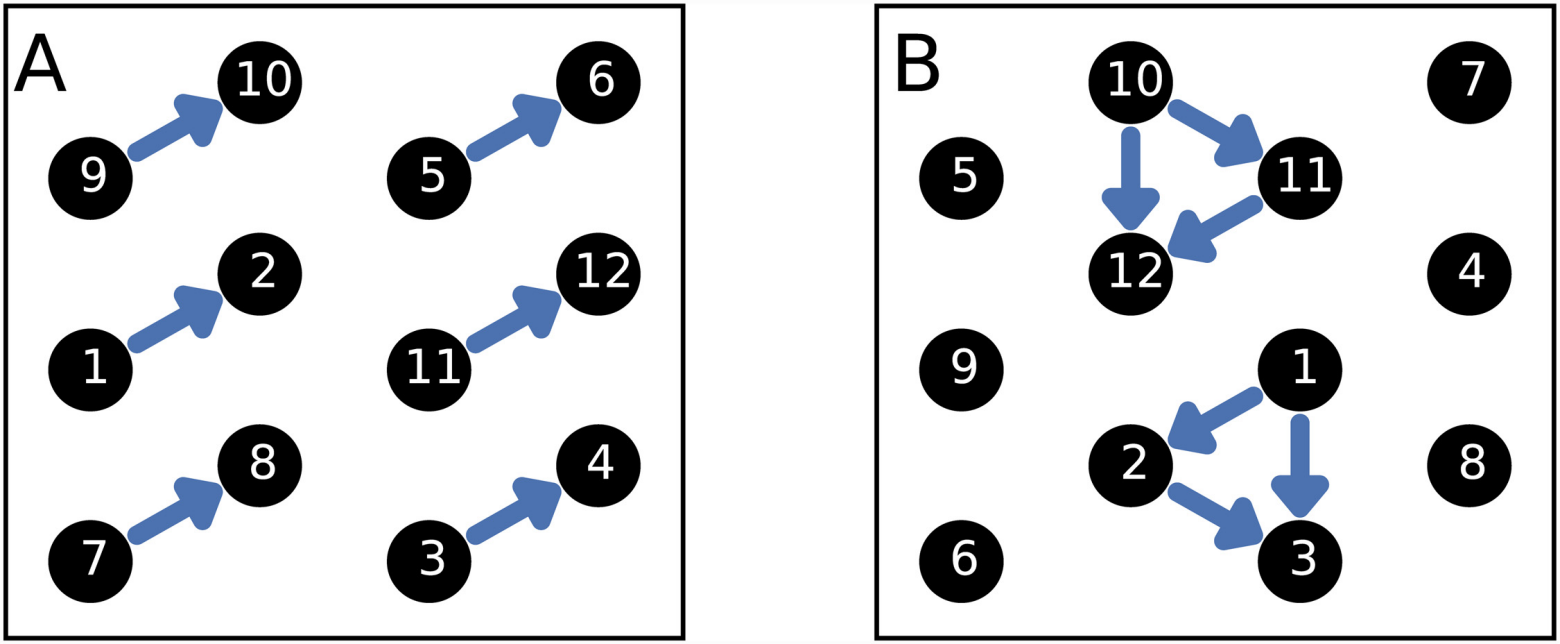
Close pair relationships for simple hypothetical sets of events. Two examples of clustering patterns are shown: in each case, the black circles show the locations of events, and their labels represent the times at which they occur. The blue arrows indicate close pairs: in this case, the temporal threshold is taken to be 2 units, and the spatial threshold is equal to the minimal separation between events. The qualitative difference between A and B results only from the permutation of temporal data.

While pair-counting methods are sufficient to establish whether clustering exists, however, they are unable to provide any insight into the particular form that it takes. The notion of clustering still allows for significant variability in the particular patterns present, as illustrated by the hypothetical examples shown in Fig 1. Both datasets contain the same number of close pairs, yet exhibit perceptible qualitative differences: Fig 1A shows a series of isolated pairs, whereas two identifiable groups are present in Fig 1B. Since the marginal spatial and temporal distributions are identical in each case—the timings have simply been permuted—existing techniques would be unable to discriminate between them.

Considering Fig 1 in greater depth, it can be seen that the essential difference between the cases is not in the number of close pairs, but rather their configuration. Any method capable of discerning between them should therefore consider the set of close pairs as a whole, and examine the relationships between them. To this end, in this paper we extend pair-based approaches by interpreting the ‘close pair’ relationship as defining an *event network*: events are represented as vertices, and edges are placed between close pairs. The analysis of the event data is thereby translated to measurement of the network’s properties, so that insight into the structure of the underlying spatio-temporal data can be gained through the application of network analysis techniques.

In particular, we consider the occurrence of ‘motifs’ within event networks. These are small subgraphs which occur disproportionately in a network (relative to an appropriate null model) and which can therefore be interpreted as the fundamental ‘building blocks’ of the network [21]. The configurations which they represent can then be reconciled with hypothesised processes driving network formation, and can offer insight into the functional role of particular structures.

Motif analysis has been applied in a number of fields. It was first used in ecology and biology [22–24] in order to identify prominent structures in, for example, protein interaction networks, gene regulatory networks and food webs. In a similar vein, neuronal networks have also been studied, with research exploring both the structure of functional circuits [25] and the dynamics of neuron firings [26]. Other real-world networks, such as transport networks [27] and those representing telecommunications links [28, 29] have also been studied in a similar way.

More recently, the approach has also been applied to data arising from dynamical systems, such as time series outputs from physical experiments [30, 31]. Networks can be derived from these outputs by forming links between output vectors on the basis of their proximity in phase space, the network thereby providing an encoding of the dynamics. Motif analysis of these networks has been used to identify characteristics in, for example, flow patterns [32–34]. Such research exemplifies the use of motif analysis as a means of encoding and characterising dynamical behaviour.

In the context of event networks, motifs represent small sets of events which occur closely in space and time in specific configurations. Their abundance therefore reveals more information than that of pairs only: while the latter might reveal that events tend to be followed closely by others, the presence of motifs can indicate, for example, whether the occurrence of two close events increases the likelihood of a third, over and above any known pair-wise dependency. In general, motifs can be interpreted as ‘signatures’ of criminal targeting processes.

Due to the nature of event networks, however, existing techniques for motif analysis cannot be applied without modification. Since their construction is geometric, event networks are constrained in the form they can take, and the edge-rewiring methods typically employed to generate randomised networks for comparison are not guaranteed to produce valid event networks. We contribute here by providing a solution to this technical challenge.

We apply these methods to real-world data concerning two types of criminal event of contrasting nature—residential burglary and maritime piracy—both of which have previously been shown to display clustering. In both cases, the analysis identifies both 3- and 4-vertex motifs, each of which can be reconciled with theories of targeting behaviour. In addition, we find that the motifs identified differ between the two crimes considered, suggesting that the method is capable of discriminating between patterns which are indistinguishable by existing approaches. In criminological terms, this highlights differences between the behavioural mechanisms at work in each case.

The primary contribution of this paper is the proposal and demonstration of a method for the measurement and characterisation of space-time clustering (that is, the nearby co-occurrence of events in space and time). The techniques provide more detailed understanding than existing approaches because, rather than simply providing a global measure of clustering, the identification of motifs highlights particular event patterns which occur prominently in the data. The methods are suitable to be applied in the analysis of any spatio-temporal point phenomenon which might display spatio-temporal association. In the case of crime (and potentially other adverse event types), the insights afforded will allow preventative activities to be targeted more precisely. Furthermore, the approach can be used to reveal that clustering patterns differ materially between event types: a fact which also has implications for the design of interventions.

## Methods

We first introduce a method for deriving networks from sets of events occurring in time and space. These *event networks* encode all information relating to the proximity of events, and provide a convenient instrument for clustering analysis. We then move on to discuss how their features can be measured statistically, whilst controlling for known clustering already present in the data.

### Event networks

In general, the data considered is assumed to consist of *N* events, indexed by *i*, each of which has some spatial location, *x*_*i*_, and a time of occurrence, *t*_*i*_. The spatial location can take any form (*e.g.* latitude/longitude or easting/northing), provided that there is some metric, *d*, for the distance between any pair of events. We will use the shorthand *d*_*ij*_ to represent the distance between two events *i* and *j*, so that

$$d_{ij}=d\left(x_{i},x_{j}\right)\geq 0.$$ (1)

Similarly, *t*_*ij*_ = *t*_*j*_−*t*_*i*_ is defined as the temporal separation between events, though this value can be positive or negative, depending on whether *j* occurs after or before *i*, respectively.

A spatial radius, *D*, and temporal radius, *T*, are taken to define what it is for two events to be ‘close’ in either dimension. These values represent parameters for the construction of the networks, and their variation will be explored later.

The close pair relationship can be used to define two networks: one, $G_{d}^{D}$, on the basis of spatial proximity, and another, $G_{t}^{T}$, on the basis of temporal proximity. In both cases, the vertices represent the events in the dataset; vertex *i* corresponds to event *i*.

$G_{d}^{D}$ is an undirected network, in which any two vertices are connected by an edge if the corresponding events occurred within a distance *D* of each other. Its edge set, $E_{d}^{D}$, is therefore

$$E_{d}^{D}=\{\left(i,j\right)|d_{ij}\leq D\}.$$ (2)

Since there is temporal ordering in the data, however, $G_{t}^{T}$ is taken to be a directed network. In this case, if two events occurred within a time window *T*, an edge is added from the earlier event to the later, and so the edge-set, $E_{t}^{T}$, is

$$E_{t}^{T}=\{\left(i,j\right)|0<t_{ij}\leq T\}.$$ (3)

It is assumed that, in general, the data are sufficiently granular that, for any pair of events, one can always be determined to have occurred first. Where this is not possible, cases where *t*_*ij*_ = 0 are resolved by deeming one event to have occurred first, at random, and directing edges accordingly.

Since they are defined by the proximity of points, both $G_{d}^{D}$ and $G_{t}^{T}$ are examples of *geometric graphs* [35]. Because of the way they are constructed, such graphs are subject to geometric constraints and require bespoke analysis [36]. A related topic concerns the properties of such graphs when the positioning of points is random [37], the crucial issue in such cases being that the randomness lies in the positioning of the points, rather than explicitly in the network itself.

From the perspective of close pairs, $G_{d}^{D}$ and $G_{t}^{T}$ carry all information about the set of events under examination. Trivially, pairs of events which are close in both space *and* time can be identified by consulting the two networks: events *i* and *j* are close in space and time if they are adjacent in $G_{d}^{D}$ and $G_{t}^{T}$. This can be formulated explicitly by defining $G_{dt}^{DT}$, the directed network of pairs that are close in both space and time, which we refer to as the *event network* of the dataset. This network is the intersection of the spatial and temporal networks, $G_{d}^{D}$ and $G_{t}^{T}$, and so its edge-set is

$$E_{dt}^{DT}=\{\left(i,j\right)|\left(i,j\right)\in E_{d}^{D}\;\text{and}\;\left(i,j\right)\in E_{t}^{T}\}.$$ (4)

### Network motifs

The objective of our analysis is to identify small subgraphs which occur disproportionately in event networks, and which can be interpreted as representing characteristic spatio-temporal patterns. Such analysis is particularly appealing when the subgraphs considered are small, since the number of possible configurations is relatively low: there are, for example, only 13 connected directed networks of 3 vertices, up to isomorphism.

**Fig 2:**
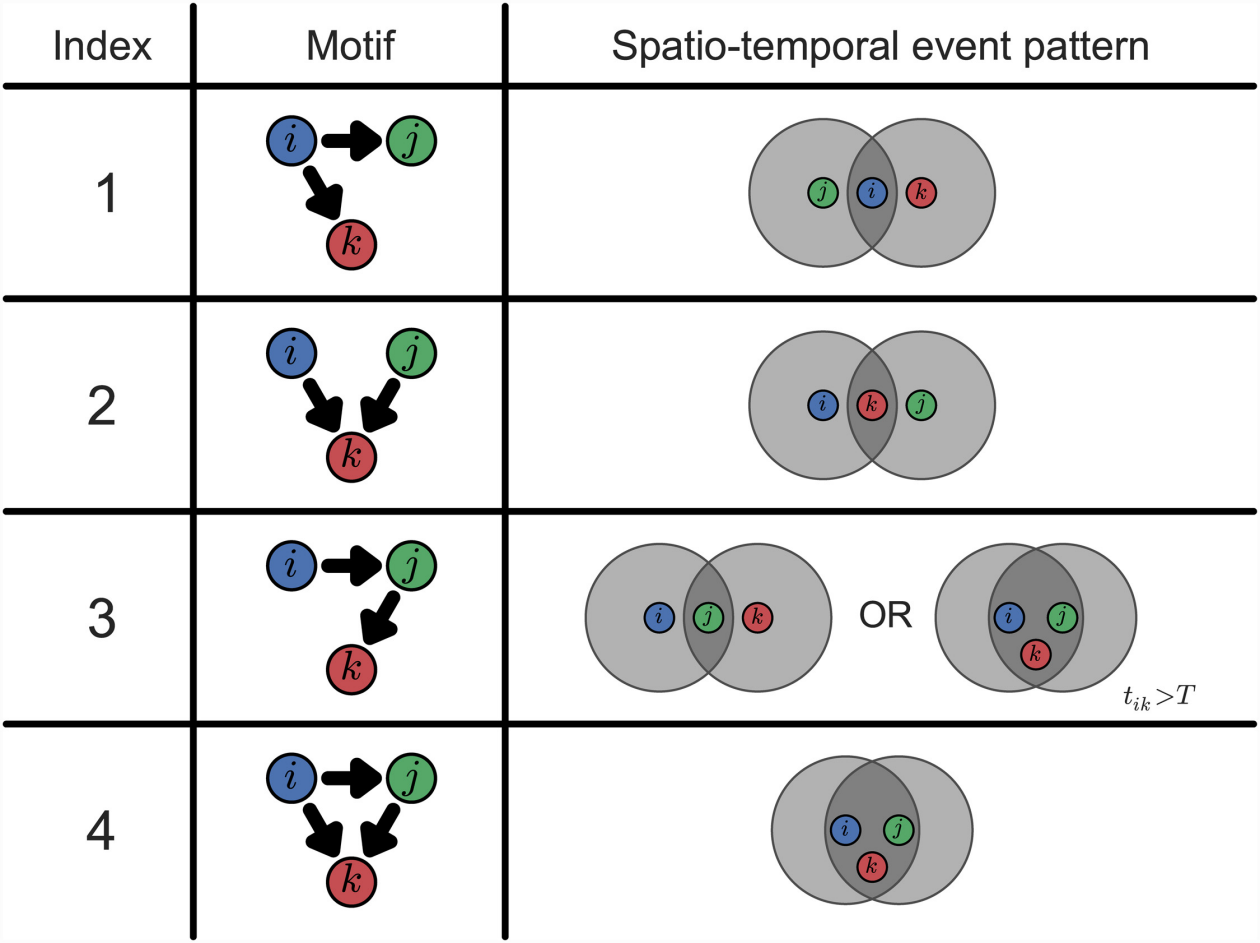
Motifs of 3 vertices and corresponding spatio-temporal patterns. All 3-vertex motifs which can arise in event networks are shown in terms of 3 generic events *i* (blue), *j* (green) and *k* (red), occurring in that order. For each motif, the spatial configuration to which it corresponds is also shown, where the shaded regions represent circular areas of radius *D*.

The number of possible motifs is reduced further still when considering event networks, as a result of the structural constraints imposed by the nature of the data. Since the direction of edges is determined by temporal data, for example, the fact that the events are temporally ordered means that any event network must be cycle-free (*i.e.* event networks are directed acyclic graphs). Such restrictions mean that only 4 motifs of order 3 (shown in Fig 2), and 24 motifs of order 4, are possible for event networks.

The ultimate value of motif analysis in this context lies in the fact that each motif corresponds to a particular spatial configuration and temporal ordering among a small group of events. The prevalence of a particular motif therefore implies that events tend to occur in the spatio-temporal pattern that it represents, and it is these patterns that constitute the ‘signatures’ of the event-set.

The patterns to which the motifs correspond can be understood in concrete terms: in Fig 2, for example, the configurations represented by each of the 3-vertex motifs are shown. In each case, the situation characterised by the motif is depicted in terms of the relationship between a triad of generic events *i*, *j* and *k* (occurring in that order), with spatial regions of radius *D* indicated.

Each pattern in Fig 2 can be understood intuitively in stylised terms: motif 1, for example, reflects dispersive behaviour, whereas motif 4 represents tight clustering. In turn, each of these can be interpreted as an expression of criminal targeting behaviour and reconciled with theory. Although we will not enumerate all possibilities here, it is clear that similar reasoning can also be applied in the 4-vertex case: each motif can be interpreted as a distinctive pattern of events.

### Statistical analysis

The most fundamental task involved in motif analysis is the counting of subgraphs of small order in a given network, and a number of well-documented methods are available for this. For sufficiently small networks, brute-force enumeration can be used (as it is here) and, in cases for which this is computationally prohibitive, efficient methods based on sampling are also available [38, 39]. The primary technical challenge of this work therefore concerns the choice of ‘random’ networks against which the observed data should be compared. Crucially, since the aim of the analysis is to gain insight beyond that offered by pair-counting methods, these reference networks should correspond to sets of events which exhibit the same level of pair-wise clustering as the observed data.

The question of which networks should be used for comparison is a fundamental one in motif analysis, and the suitability of various approaches has been the subject of debate [28, 40, 41]. In order for analysis of this type to be meaningful, some properties of the reference networks must be matched with the observed network, since otherwise the identification of motifs may be spurious: most obviously, the reference networks must have the same number of edges as the observed network, since more dense networks will naturally contain more connected subgraphs. Indeed, this highlights the direct correspondence between the choice of reference networks and the interpretation of motifs: a motif is simply a structural feature of the network that is inconsistent with the model used to generate the reference networks.

In motif analysis, the most common method of producing reference networks is to randomise the observed network by simply re-wiring its edges; that is, by re-assigning one or both of the end-points of some edges. In this way, the total number of edges is maintained, and other structural features of the network (*e.g.* vertex degrees) can also be preserved by employing variations of this approach [42]. The re-wiring process is repeated a number of times in order to generate an ensemble of randomised networks, which are taken to constitute a representative random sample of all networks which possess the prescribed properties.

This approach, however, cannot be applied in the context of event networks. As noted, the fact that such networks are derived from spatial and temporal data constrains the space of possible configurations; they must, for example, be cycle-free. Furthermore, since edges represent spatial proximity, their existence is not mutually independent, and some combinations cannot arise from any possible set of events [37]. The 9-vertex ‘star’ network is such a combination: in 2-dimensional space, it is impossible for eight points to all lie within a radius *D* of a given point without at least two of those eight being within *D* of each other themselves. An approach based on re-wiring is therefore liable to generate event networks which are invalid, in the sense that they do not represent any possible set of spatio-temporal events.

**Fig 3:**
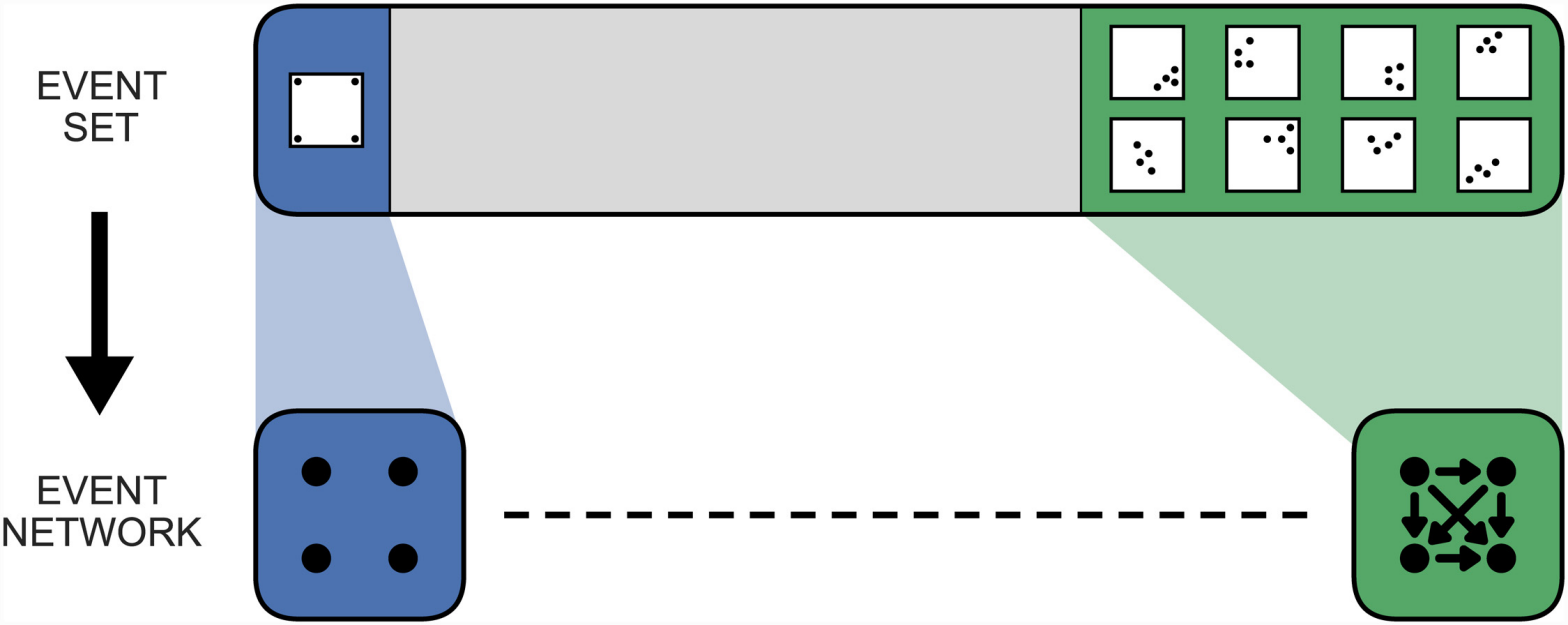
The mapping between event sets and their event networks. This schematic diagram represents the mapping between the space of event-sets and the space of event networks, for an illustrative case involving 4 events. For simplicity, the events are all assumed to be close temporal pairs, and the region is taken to be a square with width equal to the spatial close pair threshold. Only one configuration—that in which the events occur at the extremes of the region—maps to the empty network, whereas many possible event-sets map to the fully-connected network.

An even more significant issue concerns the fact that, even amongst valid networks, not all are equally likely to arise from random event data. The mapping from event-sets to their event networks is many-to-one, and the number of event-sets which give rise to each event network is not equal. Fig 3 illustrates this using a simple example of 4 events: in the scenario shown, the number of event-sets which map to the fully-connected event network is much larger than the number which map to the empty event network. This is true more generally: some event networks arise more readily than others, and therefore sampling randomly from the space of valid event networks is not equivalent to sampling from the space of event-sets. Since our ultimate interest is in the randomness (or otherwise) of the event data, sampling from the space of event networks is therefore inadequate.

### Random network generation

In light of these observations, it is clear that the generation of appropriate randomised networks cannot be done while remaining agnostic to the nature of the underlying data, and that a novel method is therefore required. Such a method should produce networks which are valid event networks (*i.e.* realisable from some set of spatio-temporal events) and which are matched with the observed event network with respect to any properties which might affect the occurrence of motifs (such as the total edge-count, for example).

The approach we take here is to explicitly simulate synthetic sets of events in time and space, constructed in such a way that the event networks derived from them have the required properties. Because events are simulated directly, the validity of the event networks is immediate, and the substance of the task is therefore concentrated on how to ensure that the generated networks possess the necessary properties. Of course, because of the duality between event-sets and their event networks, this is equivalent to producing sets of events with a prescribed level of clustering.

The most immediate property which must be matched is the total edge-count (*i.e.* the synthetic events must have the same number of close space-time pairs as the observed data). However, it is also necessary to impose further constraints. Because the event network $G_{dt}^{DT}$ is the intersection of the two proximity networks $G_{d}^{D}$ and $G_{t}^{T}$, ensuring that the edge-count of $G_{dt}^{DT}$ is maintained still permits significant variation in the character of the underlying events. The number of close spatial pairs, for example, could lie anywhere between the number of close space-time pairs and the maximum possible value of $\frac{1}{2}N\left(N-1\right)$. This clearly has potential to bias the analysis, since the event-sets being compared might be of very different character.

In order to control for this, we therefore also stipulate that the sets of synthetic events must also contain the same number of spatial and temporal pairs as the observed data. Defining the spatial, temporal and spatio-temporal networks derived from the synthetic data as ${\tilde{G}}_{d}^{D}$, ${\tilde{G}}_{t}^{T}$ and ${\tilde{G}}_{dt}^{DT}$ respectively, the requirement is therefore that each of these should have the same edge-count as its counterpart for the observed data. While the exact distributions may differ, this ensures that the synthetic events will be ‘as clustered’, in a pair-wise sense, as the observed data.

The generation of synthetic event-sets is achieved by taking a set of fully random events as a start point, and making iterative adjustments to it until the required conditions are satisfied. We begin by generating *N* events within the region in question, uniformly at random in both space and time. The number of close pairs in space, time, and both space and time are calculated for these events, and denoted ${\tilde{M}}_{d}$, ${\tilde{M}}_{t}$ and ${\tilde{M}}_{dt}$ respectively. We then define an ‘energy’ *E*, quantifying the extent to which these measurements differ from those required:

$$E=\frac{|{\tilde{M}}_{d}-M_{d}|}{{\tilde{M}}_{d}+M_{d}}+\frac{|{\tilde{M}}_{t}-M_{t}|}{{\tilde{M}}_{t}+M_{t}}+\frac{|{\tilde{M}}_{dt}-M_{dt}|}{{\tilde{M}}_{dt}+M_{dt}}$$ (5)

This energy is zero only when the required counts are equal for the observed and synthetic data, and increases as the deviation increases. The process which follows therefore repeatedly makes changes to the event set with the aim of reducing *E* until it is zero. This process, and the form of *E*, is inspired by the approach used in previous work on motif analysis in a non-spatial context [21].

**Fig 4:**
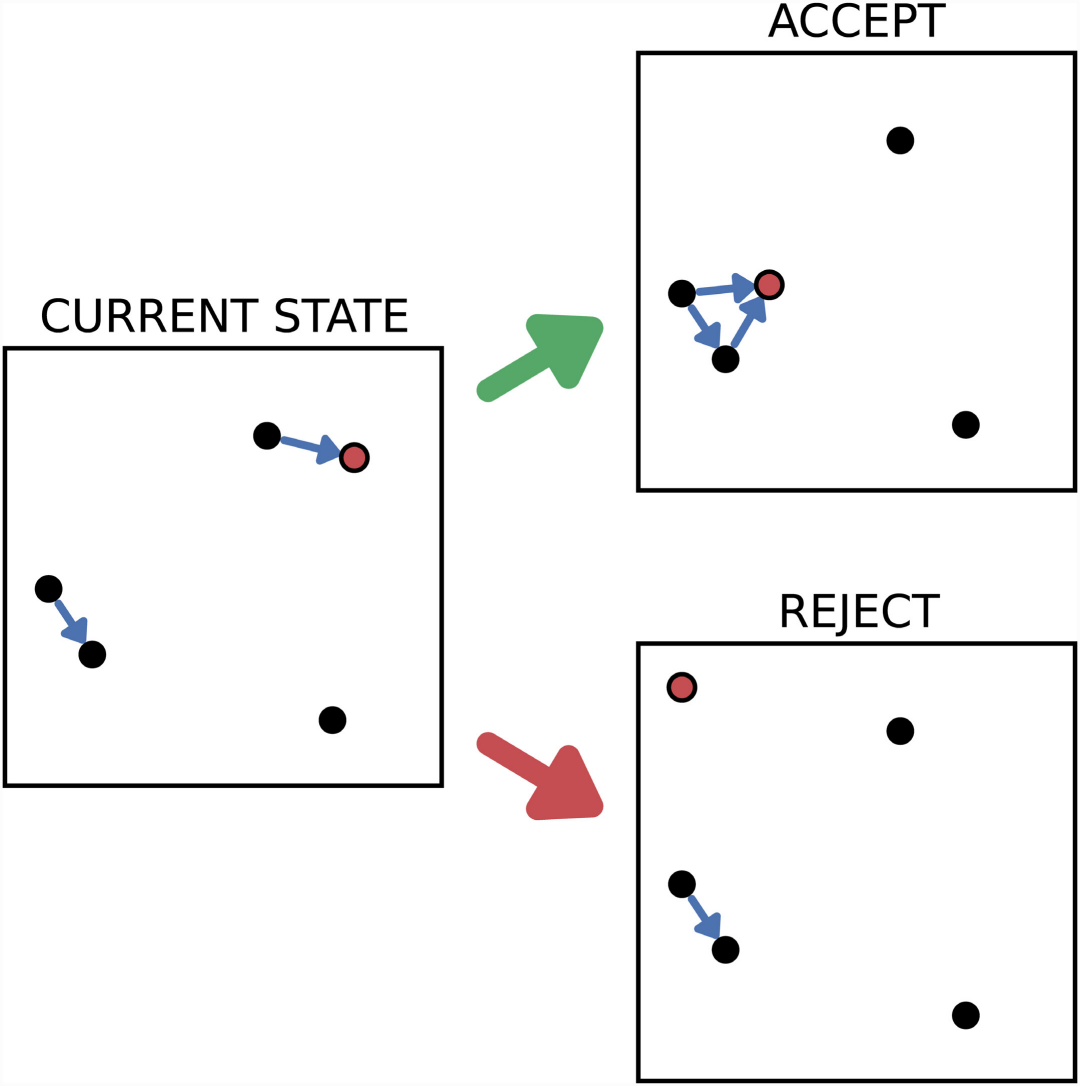
Randomised network generation. The generation of null-distributed reference networks involves iterative movement of points. The plots show the two possible outcomes at each iteration, for a hypothetical situation where 4 close pairs are required and it is assumed that all events are close temporally. The initial configuration, shown left, contains 2 close pairs. The point highlighted red is selected to move: one possible change gives 3 pairs and would be retained; the other reduces the number to 1 and would be rejected.

Each iteration involves first establishing the current energy, *E* ^*cur*^, and then selecting one of the events, uniformly at random, as a candidate for adjustment. A prospective new position and time are then generated for the selected event, chosen uniformly at random in the known spatio-temporal region. The value of the energy under this hypothetical change, *E* ^*new*^, is calculated and compared with *E* ^*cur*^. If *E* ^*new*^ < *E* ^*cur*^, the prospective change is made definite; otherwise, it is discarded and the original positions are retained (see Fig 4). In this way, *E* decreases monotonically as the algorithm iterates, and the process eventually ends when it reaches zero. A small random error can be introduced whereby some energy-increasing changes are accepted (to avoid local minima, in the spirit of simulated annealing [43]) but this is not found to be necessary in the examples considered here.

Given an observed set of events, a spatial threshold *D* and temporal threshold *T*, this method provides a means of producing synthetic sets of events with the same number of close pairs as the observed data. Since the observed and synthetic events are equivalent in terms of pair-wise clustering, yet random in all other respects, the frequency of subgraphs in the observed and synthetic event networks can be meaningfully compared.

## Results

We carry out motif analysis of event networks for two sets of real-world crime data from different domains: residential burglary and maritime piracy. Both are crime types which have previously been shown to be clustered in space and time [5, 13], but are of significantly different character in many other respects.

### Crime event data

The burglary data consists of 5,690 incidents, representing all residential burglaries recorded by police in the city of Birmingham, UK, between March 2012 and February 2013, inclusive. These are incidents which have been reported to police and officially classified as criminal events. For each incident, the location of the victimised property is recorded in terms of a British National Grid co-ordinate reference, to an accuracy of 1 metre. The temporal data takes the form of a window, representing the earliest and latest possible times at which the incident could have taken place; to establish a point estimate, we take the midpoint of these times.

The piracy data consists of the 545 pirate attacks recorded by the International Maritime Organisation as occurring during the year 2010. These records were obtained from the Anti-Shipping Activity Messages database maintained by the US National Geospatial Intelligence Agency (http://msi.nga.mil/NGAPortal/MSI.portal). The incidents include both successful and attempted attacks: since these are equivalent from the perspective of target selection, and since both require a response, both of these are included in the analysis. For each incident, the best known location is provided in terms of a latitude/longitude grid reference, and the day on which the attack occurred is also given.

### Comparing datasets

One issue to be considered when analysing these datasets in tandem is the disparity in spatial scales. The burglaries are contained entirely within an urban area, and analysis of this crime typically considers close pairs to be those which have occurred within a few hundred metres of each other [5]. Pirate attacks, on the other hand, are distributed at a global scale, and a distance of less than 100 kilometres might be regarded as ‘close’ [13].

The distances involved have different implications in either case: variation in terrain and transport modes mean that the scales are not directly comparable. Indeed, the practical implications are also different: seas are sparse environments in which policing typically involves the monitoring of large areas [44], whereas interventions concerning burglary are usually much more local [45]. In both cases, though, clustering (at whatever scale it is meaningful) has theoretical implications, and might form the empirical basis for some form of predictive approach.

The results of any clustering analysis of this form depend crucially on the choice of spatial and temporal thresholds which define a ‘close pair’. These determine the amount of information encoded in an event network: if the thresholds are too small, so few edges will exist that few details can be extracted, but if they are so high that too many edges exist, the concept of closeness is diluted and meaningful patterns might be concealed. Indeed, if analysis is carried out for several scales, the range over which significant results are found (if any) can be regarded as defining a typical scale for clustering.

In order to compare between the datasets, therefore, it is necessary to establish some equivalence between their scales from the perspective of clustering. To do this, we consider the number of close space-time pairs, relative to the number of events (in terms of the network $G_{dt}^{DT}$, the number of edges divided by the number of vertices, which is half of the mean degree). This gives an approximate measure of the concentration of edges present in the network (the usual network measure of *density* is not used, since its denominator—the maximum number of possible edges—is not well-defined for event networks). These values are given for a variety of spatial and temporal thresholds in Table 1.

**Table 1:** Concentration of edges in the event networks of each crime type.

**Temporal radius**	**Spatial radius**				
	**Burglary**				
	100m	200m	300m	400m	500m
7 days	0.10	0.21	0.36	0.54	0.74
14 days	0.15	0.33	0.60	0.92	1.29
21 days	0.18	0.44	0.82	1.29	1.83
28 days	0.22	0.56	1.05	1.66	2.36
35 days	0.26	0.68	1.27	2.02	2.87
	**Piracy**				
	50km	100km	150km	200km	250km
7 days	0.29	0.39	0.52	0.61	0.72
14 days	0.43	0.57	0.78	0.90	1.08
21 days	0.57	0.77	1.06	1.25	1.50
28 days	0.66	0.90	1.25	1.46	1.79
35 days	0.70	0.99	1.41	1.66	2.07

For each threshold combination, the value represents the ratio of the total number of close space-time pairs to the total number of events. In terms of event networks, this is equivalent to the number of edges divided by the number of vertices.

While there is substantial variation as thresholds vary, both tables contain values of broadly similar order. This suggests that the threshold values shown give rise to networks of approximately equivalent density in each case, and that they can therefore be compared meaningfully. Moreover, the presence of equal values in both tables invites direct comparison between the corresponding cases.

Before considering motifs, the data were tested for space-time clustering using the Knox permutation test [18], which measures it in a purely pair-wise sense. The test and results, both of which are described in detail in S1 Appendix, confirm that both sets of events exhibit highly significant space-time clustering, across several definitions of the close pair threshold. The values given in Table A of S1 Appendix are consistent with those published elsewhere for these crime types [5, 13].

### Empirical motif analysis

Having confirmed that clustering is present for both event types, we now investigate whether motif analysis of their event networks can offer additional insight into the patterns present. Since the analysis is performed with respect to a null model which preserves the level of pair-wise clustering in the observed data, any structures identified can be ascribed to variation in the type of clustering present, rather than its presence *per se*.

For any spatial threshold *D* and temporal threshold *T*, the first step in the analysis is to construct the various associated networks: the spatial and temporal proximity networks, $G_{d}^{D}$ and $G_{t}^{T}$, and the event network, $G_{dt}^{DT}$. The occurrences in $G_{dt}^{DT}$ of each subgraph are counted and recorded, as are the numbers of edges in all three networks.

Using the process described in *Random network generation*, an ensemble of 99 synthetic sets of events is then produced. For each of these, the event network is constructed and the frequencies of each subgraph are computed. For each subgraph, these frequencies form a null distribution against which the observed counts can be compared. Significance can be estimated by calculating the fraction, *p*, of randomised networks for which the frequency is at least as extreme as the observed count: low values of *p* therefore imply that the subgraph occurs significantly more or less frequently than would be expected under the null model. Similarly, the *z*-score of the observed count can be calculated with respect to the null distribution.

**Fig 5:**
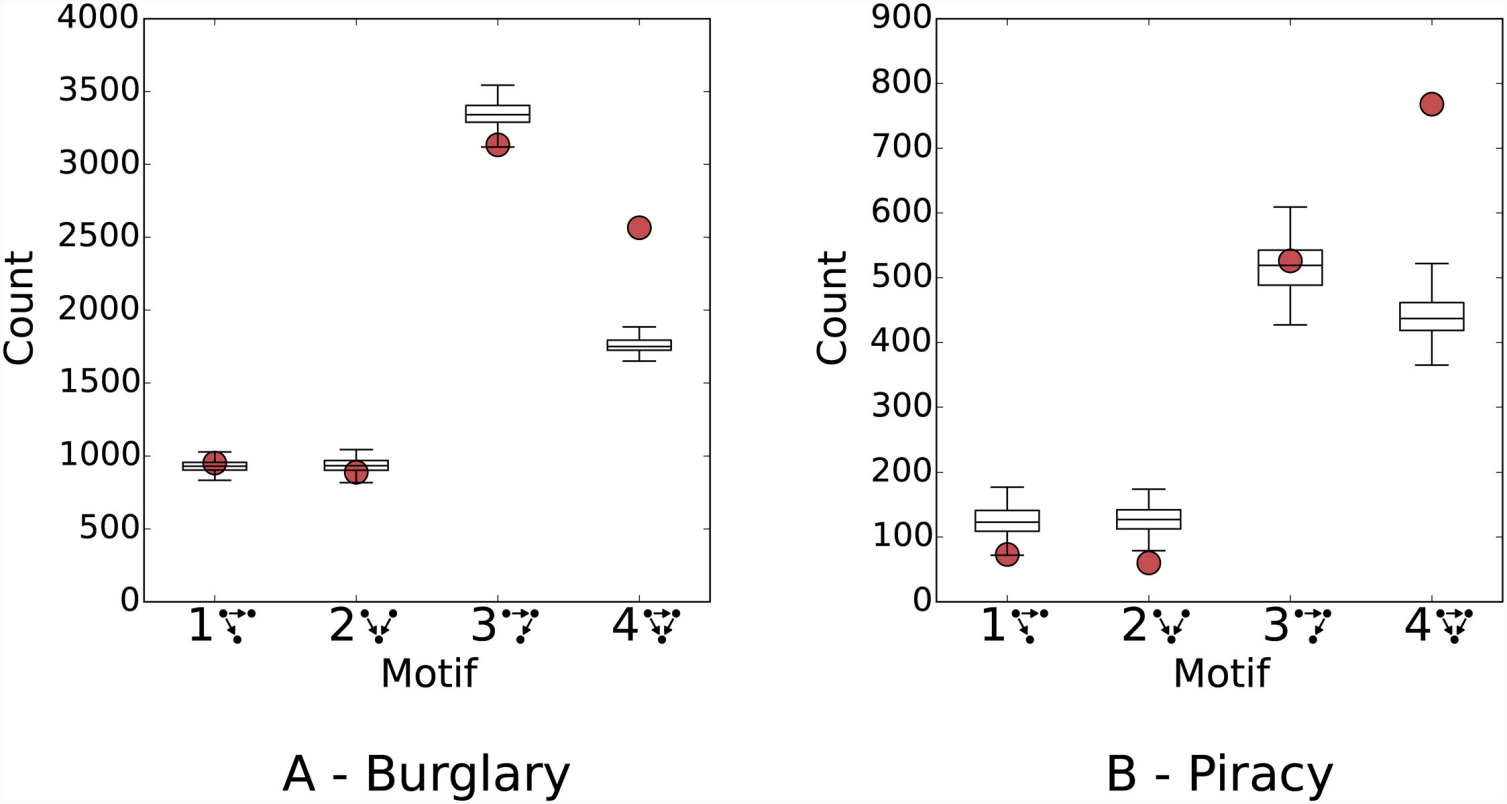
Comparison of observed and randomised counts of 3-vertex motifs. Results are shown for A) residential burglary, with a close pair threshold of 21 days and 400 metres, and B) maritime piracy, with a threshold of 21 days and 200 kilometres. Observed counts are indicated by circular markers, and box-plots give the distribution of counts across the 99 synthetic datasets (with the whiskers indicating their range).

Considering the 3-vertex case first, Fig 5 shows the results of this analysis, for particular spatio-temporal thresholds, for each of the datasets. The thresholds chosen—(21 days, 400m) for burglary and (21 days, 200km) for piracy—correspond to an approximately equal concentration of edges within the networks (see Table 1), so that they constitute equivalent scales. For each of the 4 possible motifs shown in Fig 2, the figures show box-plots of the counts within the synthetic networks, along with the observed frequency.

The most striking result in both cases is the highly significant over-representation of motif 4, which appears with a frequency far above that which would be expected on the basis of chance. That this motif—a fully-connected triple—should be so prevalent suggests that the occurrence of edges in these networks is not independent, and that they have a significant tendency to co-occur in this way. In terms of the events they represent, the result suggests that events tend to occur not just in pairs, but in fact in larger groups within a restricted spatio-temporal domain (see Fig 2). In particular, it implies that highly-clustered triples of events are prevalent in the data, with risk concentrated in a much smaller area than the circular regions typically considered.

The only other notable result for these thresholds is the under-representation of motif 2 in the piracy data (although the significance and magnitude are only marginal, the observed count is lower than all synthetic networks). This motif represents a situation in which an event occurs in the common neighbourhood of two previous events which were not themselves a close pair. This pattern can be interpreted as corresponding to a situation in which the two initial events are committed by different offenders; otherwise, if the same offender was responsible, such a pattern would imply a reversal of direction. Its absence is therefore consistent with the hypothesis that the presence of multiple active offenders in some region is unlikely.

More generally, the results can also be interpreted as a whole: the absence of significance in almost all cases for patterns other than motif 4 suggests that it is primarily configurations of that type that are responsible for the overall clustering present in the data.

**Fig 6:**
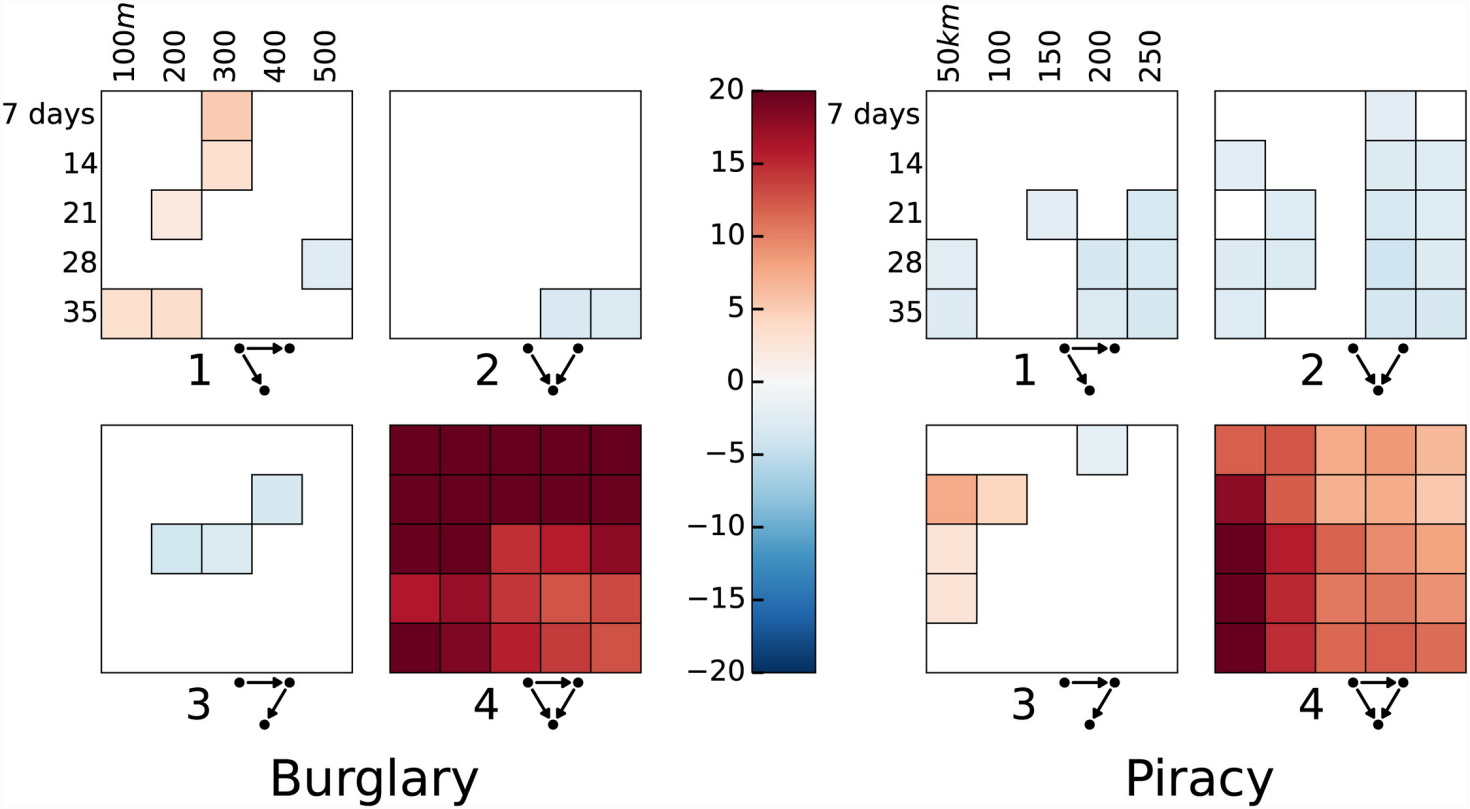
Statistical analysis of 3-vertex motifs for a variety of spatio-temporal thresholds. For any given cell, a black outline indicates that the frequency of the given motif is significant at 0.01 level; in such cases, the colour of the cell corresponds to the *z*-score, with blue indicating that the motif is under-represented and red that it is over-represented.

The results presented in Fig 5 correspond to one particular choice of *D* and *T*, but the analysis can, of course, be carried out for arbitrary choices of spatial and temporal thresholds. In Fig 6, we summarise the results for an array of threshold combinations (the same as those for which the results of the Knox test are given in S1 Appendix). For all cells for which a motif is significant at *p* = 0.01 level, the colour indicates the *z*-score for the effect.

As the threshold values vary, a number of general trends are apparent. The significance of motif 4, for example, is ubiquitous across all thresholds, suggesting again that the dense clustering to which it corresponds is a fundamental signature of both processes. In the case of piracy, its effect size is noticeably larger at smaller spatial scales but larger temporal scales, which can be interpreted as implying a characteristic scale, in some sense, for behaviour of this type. In the case of burglary, the largest effects are seen at the shortest temporal scales, which again may be instructive as to the time course of such victimisation.

In addition, significant under-representation of motifs 1 and 2 within the piracy data is apparent for a range of threshold values. A hypothetical explanation can again be offered: such motifs correspond, broadly, to more spatially-diverse offending, so that their relative absence might be suggestive of localised territorial behaviour by a small number of offenders. This accords with previous research on the topic [44].

**Fig 7:**
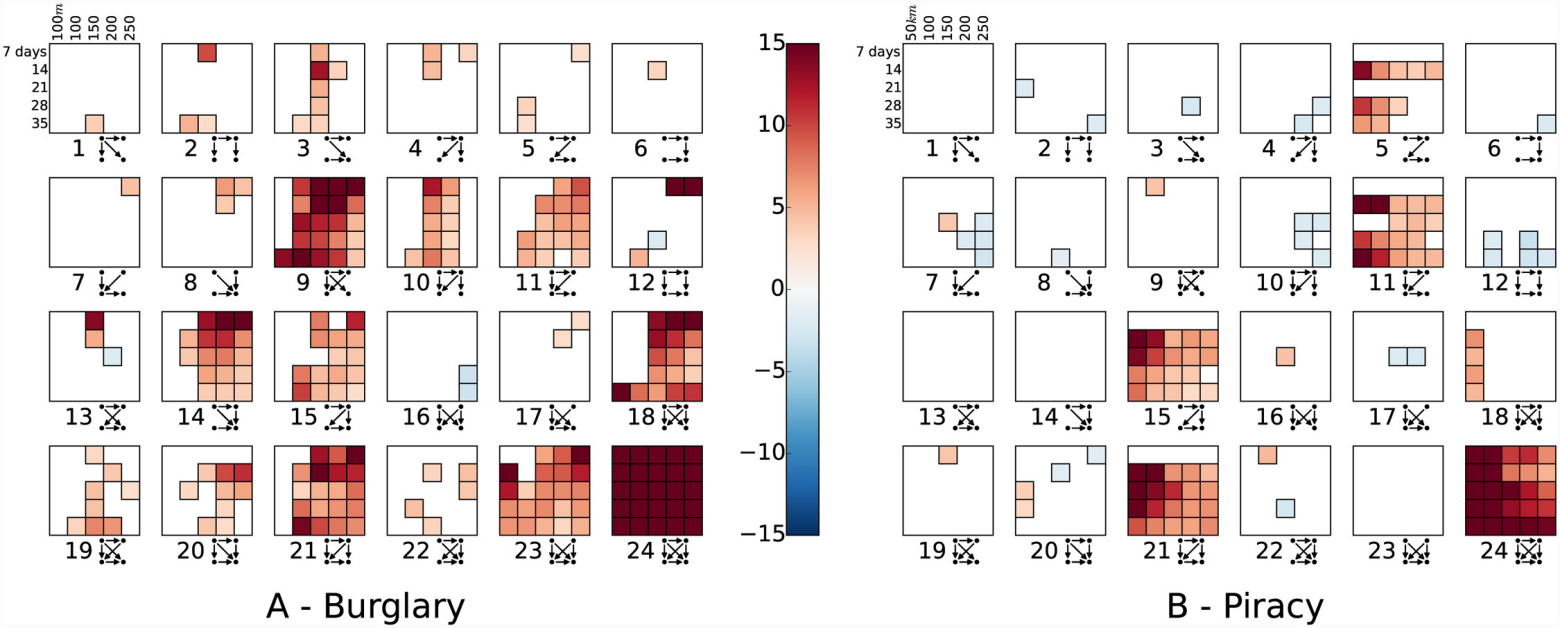
Statistical analysis of 4-vertex motifs for a variety of spatio-temporal thresholds. For any given cell, a black outline indicates that the frequency of the given motif is significant at 0.01 level; in such cases, the colour of the cell corresponds to the *z*-score, with blue indicating that the motif is under-represented and red that it is over-represented.

Moving on to consider 4-vertex subgraphs, the overall results for the same spatial and temporal thresholds as in Fig 6 are shown in Fig 7; again, several motifs are evident in each case. Indeed, a number of these, such as 11, 15, 21 and 24, show highly significant over-representation in both cases. Motif 24, the fully-connected pattern, is analogous to the fully-connected motif in the 3-vertex case, and its significance here represents further evidence of particularly dense clustering in both datasets. The other three common motifs—11, 15 and 21—are all close relations of the fully-connected 3-vertex motif, augmented in various ways by an additional event: this demonstrates that ‘drift’ behaviour is also present to some extent.

Given, the number of subgraphs presented in Fig 7 (and their close similarity), it is infeasible to provide an exhaustive exploration of each here (though there is scope for doing so). However, we suggest that the most significant result in this case is the difference between event types in terms of the motifs identified, rather than the particular patterns revealed. Although some motifs are significant in both cases, several others display contrasting behaviour. This implies that the underlying spatio-temporal patterns differ in some way, which would not be revealed by existing methods for clustering analysis.

Particularly clear examples of the disparity are motifs 9, 10, 14, 18 and 23, which are highly significant for burglary but barely so at all in the case of piracy. Indeed, several motifs are significantly under-represented for piracy, such as 7, 10 and 12. All three of those correspond to spatially-diverse patterns of events, the unlikelihood of which can be reconciled with the maritime setting: it is known, for example, that the use of spatially-anchored ‘motherships’ is common. Such territoriality is unlikely to be seen for burglary: there are likely to be multiple active offenders and the willingness to move between areas is much greater.

The higher overall number of motifs in the case of burglary also indicates a more dense, though still stylised, pattern of offending. Their presence may be influenced by the fact that the offences in question take place in a complex urban environment. In contrast to the maritime setting, this means that offender movement is constrained, and that targets are distributed non-uniformly. This may explain the presence of less densely-connected motifs.

Overall, however, the most crucial observation in the 4-vertex case is simply the disparity between event types in terms of the patterns identified. This suggests that analysis of this type is capable of revealing differences in the patterning of offences at a deeper level than previously possible.

## Discussion

The question of whether a set of events, embedded in time and space, is clustered is a fundamental one in many disciplines, and is of particular significance in the study of crime. Existing statistical tests are based on the pair-wise comparison of events, and identify situations in which the co-occurrence of events is more (or less) frequent than would be expected by chance. While these are sufficient to identify clustering *per se*, however, their treatment of pair-wise relationships as independent occurrences means they are unable to identify more subtle underlying patterns.

In this work, we have demonstrated how the proximity of events can be encoded as a graph, called an *event network*, the features of which can then be analysed in order to gain insight into the spatio-temporal data. While existing approaches can be formulated in these terms, the network approach represents a substantial refinement since it allows more complex network features (and therefore patterns) to be examined. In this paper, we have focussed on the identification of motifs: small subgraphs which represent the building blocks of the network and can be interpreted as ‘signature’ space-time patterns.

In order to identify motifs, the observed network must be compared against a suitable null comparison. In this case, since the aim is to identify patterns over and above the pair-wise clustering already known, it is necessary to generate networks which are equivalent to the observed data in terms of clustering. With this in mind, we introduced a novel approach by which synthetic sets of events can be generated so as to have a prescribed level of clustering. This technique is likely to be of wider use in providing null models for the statistical analysis of spatio-temporal data.

Application of these techniques to data for two contrasting crime types—residential burglary and maritime piracy—reveals significant motifs of 3 and 4 vertices. Significant over-representation of the most highly-connected 3-vertex subgraph is seen for both datasets, from which it can be inferred that not only do events tend to co-occur, but that close pairs of events themselves tend to group together in a highly dense manner. This accords with theories such as the optimal forager principle, which states that offenders repeatedly return to the same area, and has clear implications for crime prevention.

Significant motifs are also seen in the 4-vertex case, again reflecting high levels of clustering. Intriguingly, however, and in contrast to the 3-vertex case, results differ markedly between the two crime types, in terms of the particular motifs identified. This is a crucial outcome from the perspective of the method itself, since it suggests that it can be used to identify differences between the patterns; traditional analysis, on the other hand, would simply show that both are clustered. Since the method controls for purely pair-wise clustering, the implication of the result is that the agglomeration of pairs into larger clusters can take different forms, which is consistent with the presence of distinct targeting mechanisms. From a practical perspective, this suggests that no single preventative strategy will be applicable in all situations where clustering is present: rather, it may need to be adapted to particular contexts.

Translating into spatio-temporal terms, the results imply that events tend to occur in the overlapping spatio-temporal vicinity of previous events. This has immediate practical implications: this overlapping region, which will typically be substantially smaller than the circular region which would be identified for a single event, experiences an additional elevation of risk. In a sense, risk acts additively in this region, and so a restricted area of ‘super-risk’ is present: this would be an appealing, and well-defined, target for policing activity.

Pair-wise clustering analysis has inspired several methods of crime prediction based on the identification of a radial region around an initial event, in which a follow-up incident might be expected to occur. The motif analysis presented here, however, raises the possibility that these techniques could be refined by predicting on the basis of groups of events, rather than individual incidents. 3-vertex motifs, for example, correspond to situations in which a close pair of events has occurred and a third can be predicted to happen in one of the surrounding regions (which, crucially, may have much smaller area than the corresponding radial region). More generally, since each motif corresponds to a particular configuration in space-time, the relative likelihood of each motif could be interpreted as the relative risk of a further event occurring in each surrounding region. Incorporating these ideas within predictive tools may allow more sophisticated predictions to be made, and this will be the subject of further research.

There are a number of ways in which the approach presented here could be refined, with respect to both the techniques themselves and their potential uses. There is clear scope, for example, for the investigation of network features other than motifs. The key theoretical innovation—the production of randomised event sets—provides a baseline against which any network property can be compared, several of which might correspond to spatio-temporal phenomena of interest.

Finally, although this paper was concerned with patterns of crime, the methods themselves are generic and could be applied in a number of other fields. A natural candidate for this is epidemiology, in which space-time clustering can be taken as evidence of contagion. In such a context, results of the kind presented here would correspond to the characterisation of transmission patterns, and might offer a means of differentiating between the behaviours of distinct infective processes. Just as motif analysis itself has been applied in a number of unrelated domains, these techniques can be used to identify signature patterns in any systems which gives rise to spatio-temporal events. Future work will examine the application of the methods to alternative topics such as these.

## Supporting Information

S1 Appendix Analysis of space-time clustering using the Knox test. A technical description of the Knox test, which measures space-time clustering in a pair-wise sense, is given. The results of this test are then presented for the two crime types considered in this paper.
(PDF)
