# Supplementary material for: Event Networks and the Identification of Crime Pattern Motifs
Source: PLoS One. 2015 Nov 25;10(11):e0143638. doi: 10.1371/journal.pone.0143638 (PMC4659661; doi:10.1371/journal.pone.0143638)
Supplement: S1 Appendix — A technical description of the Knox test, which measures space-time clustering in a pair-wise sense, is given. The results of this test are then presented for the two crime types considered in this paper. (PDF) [file pone.0143638.s001.pdf]

S1 Appendix for 'Event networks and the identification of crime pattern motifs'

Knox clustering analysis

In order to establish whether the events considered in the paper were indeed clustered in a traditional, pair-wise sense, a Knox-type test was performed on the two datasets. In the form used here, this is a permutation test, for which the null hypothesis is that the close proximity of events in space is independent of that in time.

The test is performed on the basis that a 'close pair' of events is one which occurs within a distance D and time T of each other. The first step is to compare every possible pair of events in the dataset and count the number of these which are close pairs, denoted S. Then, the locations of the events are permuted (*i.e.* shuffled) while the times of occurrence remain constant: if this permutation is σ, then an event i occurring at (t_i, x_i) is mapped to $(t_i, x_{\sigma(i)})$. The number of close pairs is then computed for these events. This process of permutation and pair-counting is repeated K times, and the set of counts of close pairs forms a reference distribution.

The statistical significance of the observed count S can then be found by comparison with this distribution. If the K pair counts are placed in an ordered list and R taken to be the rank at which S would be placed within this list, a p-value is given by

$$p = \frac{R}{1+K}. \qquad (1)$$

In addition, the magnitude of any effect can be measured by computing a z-score for S, relative to the reference distribution. A higher z-score corresponds to a greater tendency for events to occur in close pairs, relative to what would be expected if spatial and temporal distributions were independent.

One particularly appealing feature of this test is that, because of the permutation approach, the underlying spatial and temporal data remains constant throughout. That is, at each permutation, the distributions in each dimension are the same as in the observed case. This has the effect of controlling for heterogeneity in either of these distributions and ensures that results are not simply an artefact of this.

This test was carried out with $K = 99$ for both burglary and piracy datasets, for a number of values of D and T in each case. In all cases, the events were found to be clustered with a p-value

of 0.01. Table A gives the z-scores for each of these. As expected, the magnitude of the effect decreases as granularity is lost, suggesting that the effects are localised in both space and time. The effect is slightly larger in the case of piracy, though the disparity between crimes is not particularly large, and the overall conclusion is that the events are highly clustered, by this measure, in both cases.

	100m	200	300	400	500
7 days	16.00	12.92	12.60	12.61	14.25
14	13.03	11.76	9.24	9.99	9.33
21	10.61	9.90	7.91	9.25	9.01
28	8.64	9.17	9.65	8.00	9.91
35	9.98	10.32	8.70	9.56	9.25

(a) Residential burglary

	50km	100	150	200	250
7 days	13.51	12.88	13.82	13.65	13.84
14	12.45	10.71	10.68	11.89	11.99
21	11.97	10.46	11.10	12.53	10.92
28	11.23	9.88	9.93	9.86	9.33
35	9.42	6.47	9.55	7.93	7.94

(b) Maritime piracy

Table A: Knox analysis: values shown are z-scores for the observed number of close pairs, relative to those found under the randomised distribution. In all cases the observed value was higher than that for all randomised datasets, corresponding to a p-value of 0.01.
